# *FLOURY ENDOSPERM16* encoding a NAD‐dependent cytosolic malate dehydrogenase plays an important role in starch synthesis and seed development in rice

**DOI:** 10.1111/pbi.13108

**Published:** 2019-03-27

**Authors:** Xuan Teng, Mingsheng Zhong, Xiaopin Zhu, Chunming Wang, Yulong Ren, Yunlong Wang, Huan Zhang, Ling Jiang, Di Wang, Yuanyuan Hao, Mingming Wu, Jianping Zhu, Xin Zhang, Xiuping Guo, Yihua Wang, Jianmin Wan

**Affiliations:** ^1^ State Key Laboratory for Crop Genetics and Germplasm Enhancement Jiangsu Plant Gene Engineering Research Center Nanjing Agricultural University Nanjing China; ^2^ National Key Facility for Crop Resources and Genetic Improvement Institute of Crop Science Chinese Academy of Agricultural Sciences Beijing China

**Keywords:** floury endosperm mutant, starch synthesis, grain weight, malate, energy supply, redox regulation, rice

## Abstract

Starch is the most important form of energy storage in cereal crops. Many key enzymes involved in starch biosynthesis have been identified. However, the molecular mechanisms underlying the regulation of starch biosynthesis are largely unknown. In this study, we isolated a novel floury endosperm rice (*Oryza sativa*) mutant *flo16* with defective starch grain (SG) formation. The amylose content and amylopectin structure were both altered in the *flo16* mutant. Map‐based cloning and complementation tests demonstrated that *FLO16* encodes a NAD‐dependent cytosolic malate dehydrogenase (CMDH). The ATP contents were decreased in the mutant, resulting in significant reductions in the activity of starch synthesis‐related enzymes. Our results indicated that FLO16 plays a critical role in redox homeostasis that is important for compound SG formation and subsequent starch biosynthesis in rice endosperm. Overexpression of *FLO16* significantly improved grain weight, suggesting a possible application of *FLO16* in rice breeding. These findings provide a novel insight into the regulation of starch synthesis and seed development in rice.

## Introduction

Starch, large biopolymers of glucose, is the most important form of carbohydrates for most organisms. As the major source of daily carbon intake for humans, starch has a vital role in our diet and health. Cereal endosperm accumulates high levels of starch that provides energy for seed germination and early seedling development. In rice (*Oryza sativa*), endosperm starch forms insoluble particles referred to as starch grains (SGs) in the amyloplasts. A SG is generally composed of a complex of sharp‐edged polyhedral starch granules (Jane *et al*., 1994), also named as compound SG (Tateoka, 1962). SGs are easily observed by staining with iodine solution using a light microscope.

Starch synthesis begins with the enzyme ADP‐glucose pyrophosphorylase (AGPase) catalysing the reaction of glucose 1‐phosphate (G1P) and ATP to ADP‐glucose (ADPG), the substrate for starch synthesis (Martin and Smith, 1995). In contrast to most higher plants and leaves of cereal crops (Beckles *et al*., 2001; Tetlow *et al*., 2003), the major forms of AGPase in cereal endosperm are located in the cytosol (Tetlow *et al*., 2003). Synthesized ADPG is then transported from the cytosol to amyloplasts by the ADPG transporter, Brittle 1 (Li *et al*., 2017; Sullivan *et al*., 1991). Five starch synthase (SS) isoforms synthesize and elongate glucan chains using ADPG as the substrate. Granule‐bound starch synthase (GBSS) acts in biosynthesis of amylose and extra‐long unit chains of amylopectin in rice (Hanashiro *et al*., 2008), and other SS isoforms (SSI, SSII, SSIII and SSIV) participate in amylopectin biosynthesis (Nakamura, 2002). Branching enzymes (BEs) and debranching enzymes (DBEs) are required to define amylopectin structure (Ball *et al*., 1996). In addition to the enzymes mentioned above, additional factors indirectly regulate starch synthesis. For example, Du1 regulates starch synthesis by assisting the splicing of *waxy* pre‐mRNA (Isshiki *et al*., 2000). Previous study in wheat revealed that protein phosphorylation regulates activity and integrity of a protein complex formed by BEIIb, BEI and plastidial phosphorylase (PHO1) (Tetlow *et al*., 2004). Evidence for interaction between BEII, SSI and SSII was also obtained in developing wheat endosperm (Tetlow *et al*., 2008). However, detailed starch biosynthesis mechanisms, especially their regulation, are far from complete resolution.

Opaque‐kernel mutant phenotypes indicate changes in storage metabolites, varying starch content and structure, aberrant SGs, and other abnormalities. Various rice mutants with opaque endosperm and individual SGs were named floury, including *flo(a)* (Qiao *et al*., 2010), *flo2* (She *et al*., 2010), *flo3* (Nishio and Iida, 1993), *flo4* (Kang *et al*., 2005), *flo5* (Ryoo *et al*., 2007), *flo6* (Peng *et al*., 2014), *flo7* (Zhang *et al*., 2016) and *flo8* (Long *et al*., 2017). Some mutants with abnormal SGs were named as *substandard starch grain* (*ssg1‐6*) (Matsushima *et al*., 2010, 2014, 2016), *dull* (*du1‐3*) (Isshiki *et al*., 2000, 2008; Zeng *et al*., 2007) and *chalk5* (Li *et al*., 2014). All previous studies confirmed that defective endosperm mutants are valuable genetic resources for dissecting the mechanisms of amyloplast development and starch biosynthesis.

Malate content contributes to redox homeostasis in the cytosol. Malate participates in the transport of redox equivalents among cell compartments (Kromer and Scheibe, 1996; Scheibe, 2004). In tomato, malate was identified as a potential metabolite regulating starch synthesis. Further analysis suggested that an altered plastidial redox status caused by modified malate metabolism resulted in different AGPase activation states and final starch contents (Centeno *et al*., 2011). Similar conclusions were made for potato (Tiessen *et al*., 2002). However, in potato tubers, starch synthesis in plastids was not affected by decreased synthesis of malate in mitochondria, suggesting that redox regulation is tissue dependent (Szecowka *et al*., 2012). However, the role that malate metabolism plays in starch synthesis in rice endosperm remains unknown.

In this study, a floury mutant, *flo16*, with a deletion of 4 base pairs in the *Cytosolic Malate Dehydrogenase* gene was isolated and characterized. Compared to the wild type, decreased starch and increased sucrose contents in the *flo16* mutant revealed that the transition from sucrose to starch was partially disrupted during mutant grain filling. Endosperm‐specific overexpression of *FLO16* led to increased grain weight. Our results demonstrated that FLO16 is important for starch biosynthesis and seed development in rice.

## Results

### *flo16* endosperm is defective in starch accumulation

**Figure 1:**
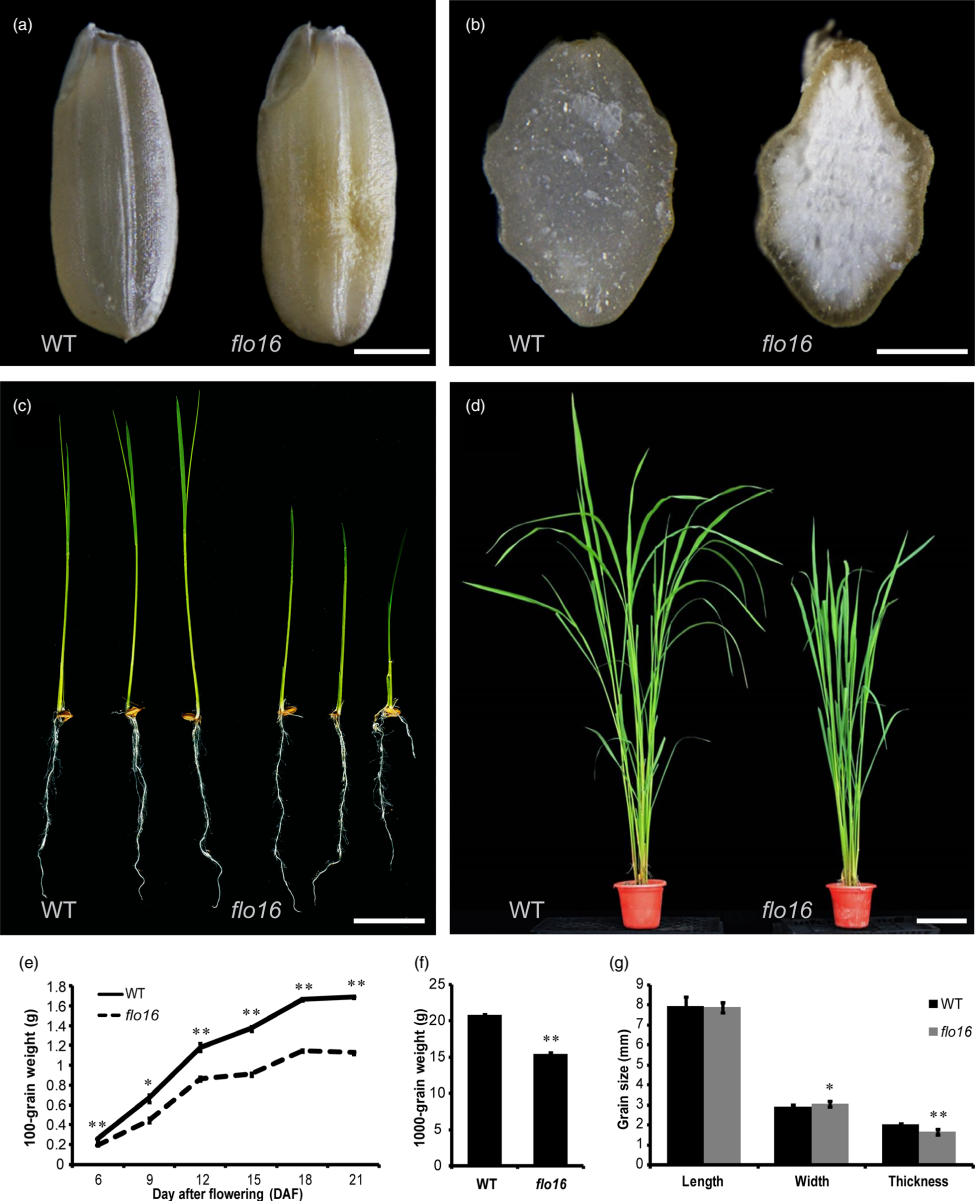
Phenotypes of the *flo16* mutant. (a) Images of mature seeds of wild type (left) and *flo16* (right). Scale bar, 2 mm. (b) Cross sections of the mature seeds of wild type (left) and *flo16* (right). Scale bar, 1 mm. (c) One‐week‐old seedlings of wild type (left) and *flo16* (right). Scale bar, 4 cm. (d) Wild type (left) and *flo16* (right) plants at the booting stage. Scale bar, 20 cm. (e) Grain weights of wild type and *flo16* at various stages post‐fertilization. Grain weight indicates the weight of 100 dehulled dry grains, values are means ± SD,* n *= 3. (f) 1000‐grain weights of wild type and *flo16*. Values are means ± SD,* n *= 5. (g) Grain size comparisons between wild type and *flo16*. Values are means ± SD,* n *= 10. Asterisks indicate the statistical significance between the wild type and mutants determined by Student's *t*‐tests (*P < 0.05; **P < 0.01).

As part of our ongoing effort to dissect mechanisms underlying starch biosynthesis and its regulation in rice, we isolated a floury endosperm mutant named *flo16* from a ^60^Co‐irradiated M_2_ population of *indica* rice variety N22. The mature grains of *flo16* were opaque and slightly shrunken compared with the transparent and fully developed grains of wild type (Figure 1a,b). During endosperm development, the *flo16* mutant underwent a much slower grain‐filling rate from 6 days after flowering (DAF), and this difference was maintained until maturation (Figure 1e). The 1000‐grain weight of *flo16* was significantly lower than that of wild type (Figure 1f); and the grain thickness of *flo16* was smaller than that of the wild type (Figure 1g). Clearly, the *flo16* mutation affects starch accumulation during endosperm development. It is worth noting that the plant height of the *flo16* mutant was also shorter in height and had less tillers compared to wild type (Figure 1c,d; Table S1). These results suggest that the *flo16* mutant is also defective in plant growth and development and that changes in starch metabolism extend beyond the endosperm.

### *flo16* endosperm has irregular starch grain morphology

**Figure 2:**
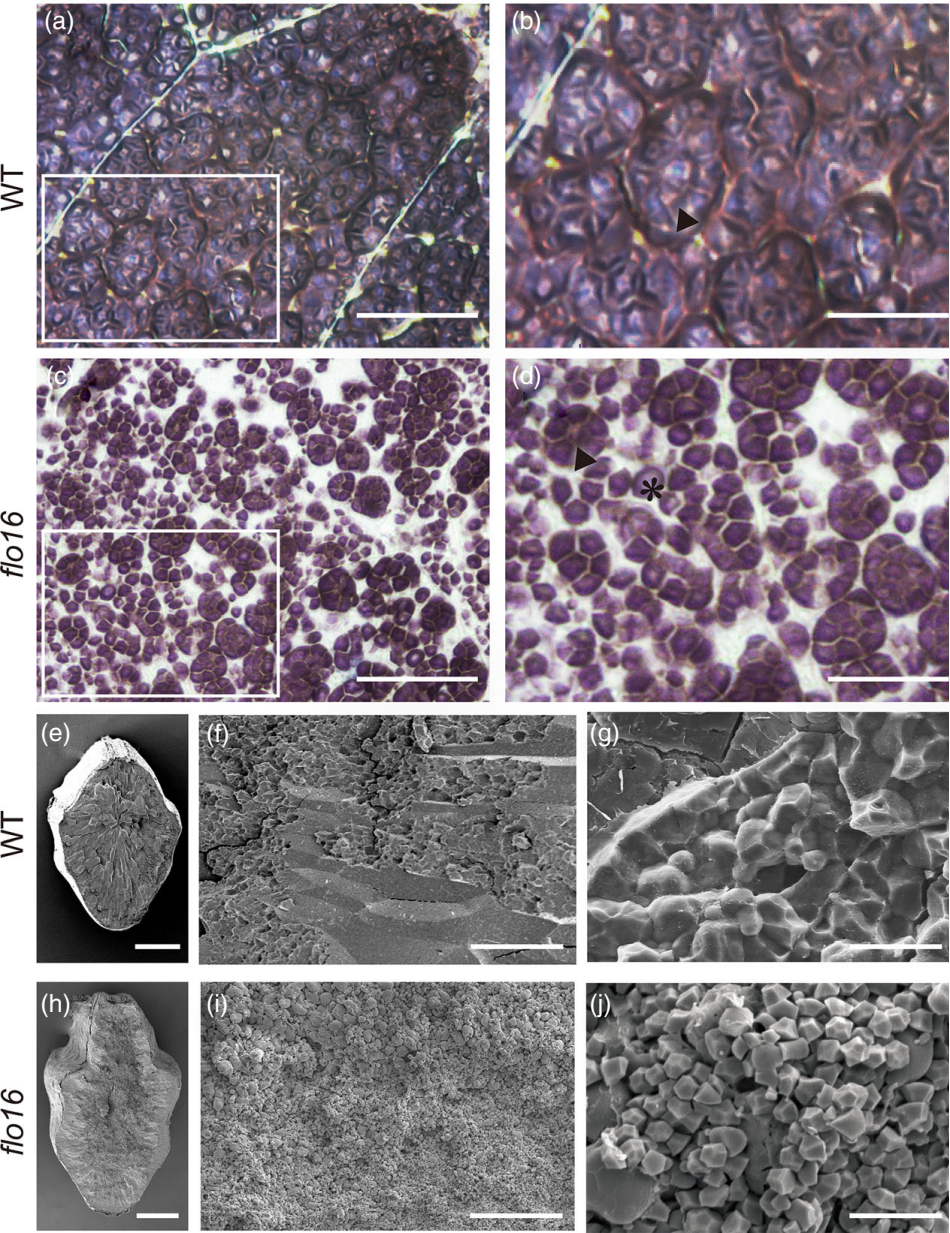
Abnormal starch grain (SG) formation in *flo16* endosperm. (a‐d) Semi‐thin sections of the wild type (a, b) and *flo16* (c, d) endosperms at 12 days after flowering (DAF). (b) and (d) are enlargements of the boxed areas in (a) and (c), respectively. Small and scattered SGs are displayed in lower left of (b, d). Triangles indicate compound starch grains in (b) and (d); a asterisk indicates a single starch grain in (d). (e–j) SEM of endosperm of wild type (e‐g) and *flo16* (h‐j). Scale bars, 40 μm in (a, b), 20 μm in (c, d), 1 mm in (e, h); 200 μm in (f, i); 10 μm in (g, j).

Semi‐thin sections of developing endosperm in *flo16* at 12 DAF examined after iodine staining had massive unstained spaces apparently caused by delayed filling of amyloplasts (Figure 2c,d). Amyloplasts in wild‐type endosperm were full of densely packed compound SGs consisting of polyhedral granules (Figure 2a,b), whereas in *flo16* endosperm they were tiny and disordered (Figure 2c,d). Besides compound SGs, many single granules were scattered in the mutant endosperm (Figure 2d). Scanning electron microscopy (SEM) of transverse sections of mature endosperm revealed that, unlike the regular, compact crystal structure of wild‐type SGs (Figure 2e–g), the endosperm of *flo16* consisted of small, spherical and loosely packed SGs with large air spaces (Figure 2h–j). Therefore, formation of compound SGs was severely disrupted in developing *flo16* endosperm.

### Physicochemical properties of *flo16* starch

**Figure 3:**
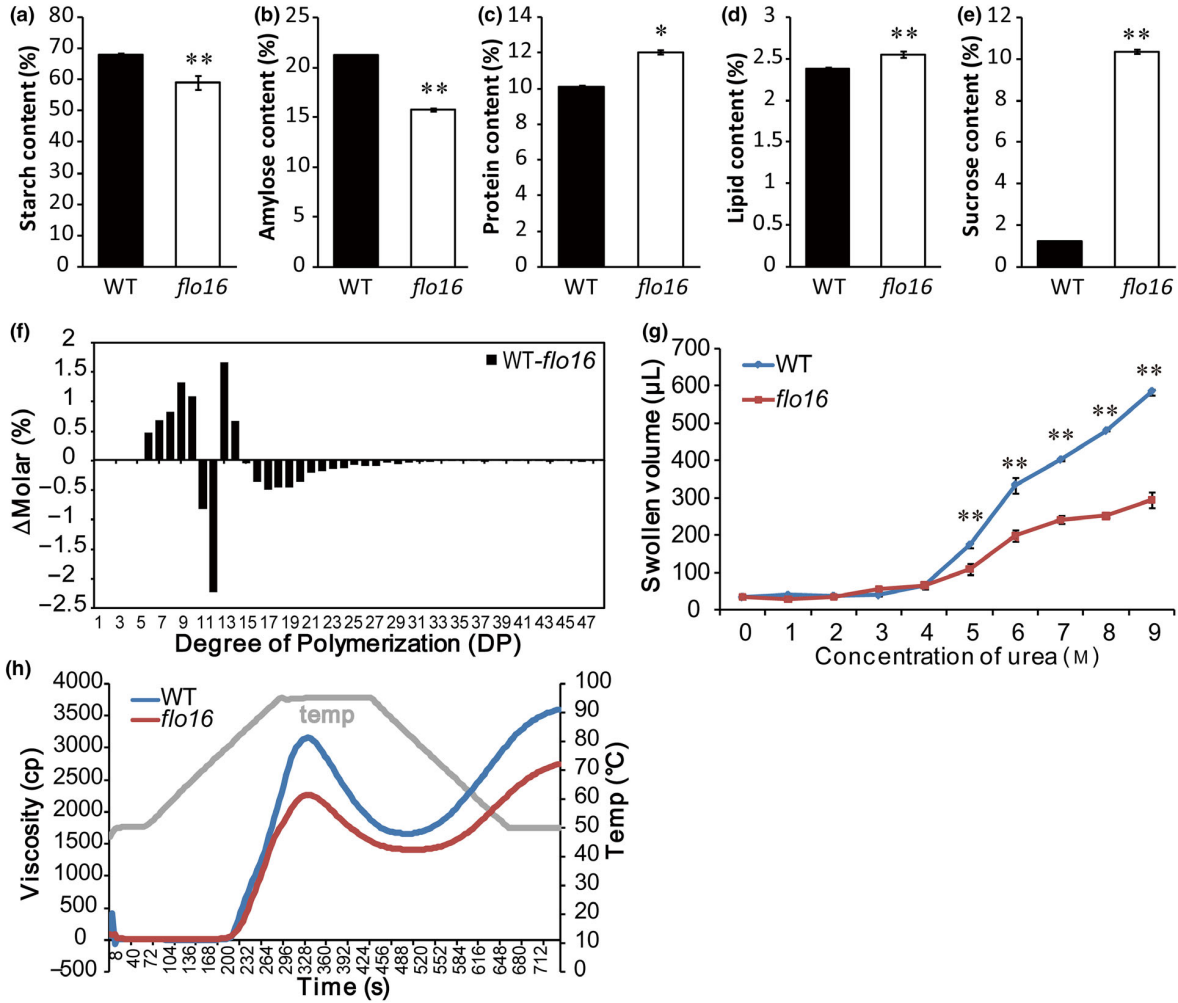
Properties of grains and physicochemical characteristics of starch in the *flo16* mutant. (a‐e) The contents of total starch (a), amylose (b), protein (c), lipid (d) and sucrose (e) were proportions of grain dry weight in wild type and *flo16*. Values are means ± SD,* n *= 3. (f) Differences in amylopectin chain length distributions between the wild type and the *flo16* mutant. (g) Swollen volumes of wild‐type and *flo16* starch in urea solutions at various concentrations (0–9 M). Values are means ± SDs, *n *= 3. (h) Pasting properties of endosperm starch of the wild type and *flo16* mutant. Grey line indicates temperature changes during measurements. Asterisks indicate the statistical significance between the wild type and mutant determined by Student's *t*‐tests (*P < 0.05; **P < 0.01).

Total starch and amylose contents in dry weight of mature *flo16* seeds were significantly less than in wild‐type seeds (Figure 3a,b), whereas protein and lipid contents were both elevated (Figure 3c,d). Sucrose content in *flo16* endosperm was 8 times higher than in the wild type (Figure 3e), suggesting disruption of transition from sucrose to starch. Although amylopectin content taken up in mature *flo16* seeds was almost unchanged, the degrees of polymerization (DP) in the ranges 6–10 and 13–14 were significantly increased, whereas those in the ranges of DP 11‐12 and DP 15‐48 were decreased (Figure 3f). There were no visible alterations in the amounts of A‐chains with DP 6‐12 and B‐chains with DP > 36 as classified by Hanashiro *et al*. (2002). However, elongation of both A‐chains and B‐chains was abnormal in the *flo16* mutant (Figure 3f). The solubility of starch in urea solution was measured to test the gelatinization properties (Nishi *et al*., 2001). Even in 9 M urea powdered *flo16* starch was difficult to gelatinize, whereas wild‐type starch began to gelatinize in 5 M urea and was thoroughly gelatinized in 9 M urea (Figure 3g). Analyses of pasting properties revealed that the viscosity of *flo16* pasting starch was low after the rise in temperature, but the curve patterns showed no difference between wild type and *flo16* (Figure 3h). Collectively, the physicochemical properties of *flo16* starch were significantly altered compared with the wild type.

### The *flo16* mutation occurred in gene *Os10 g0478200*

**Figure 4:**
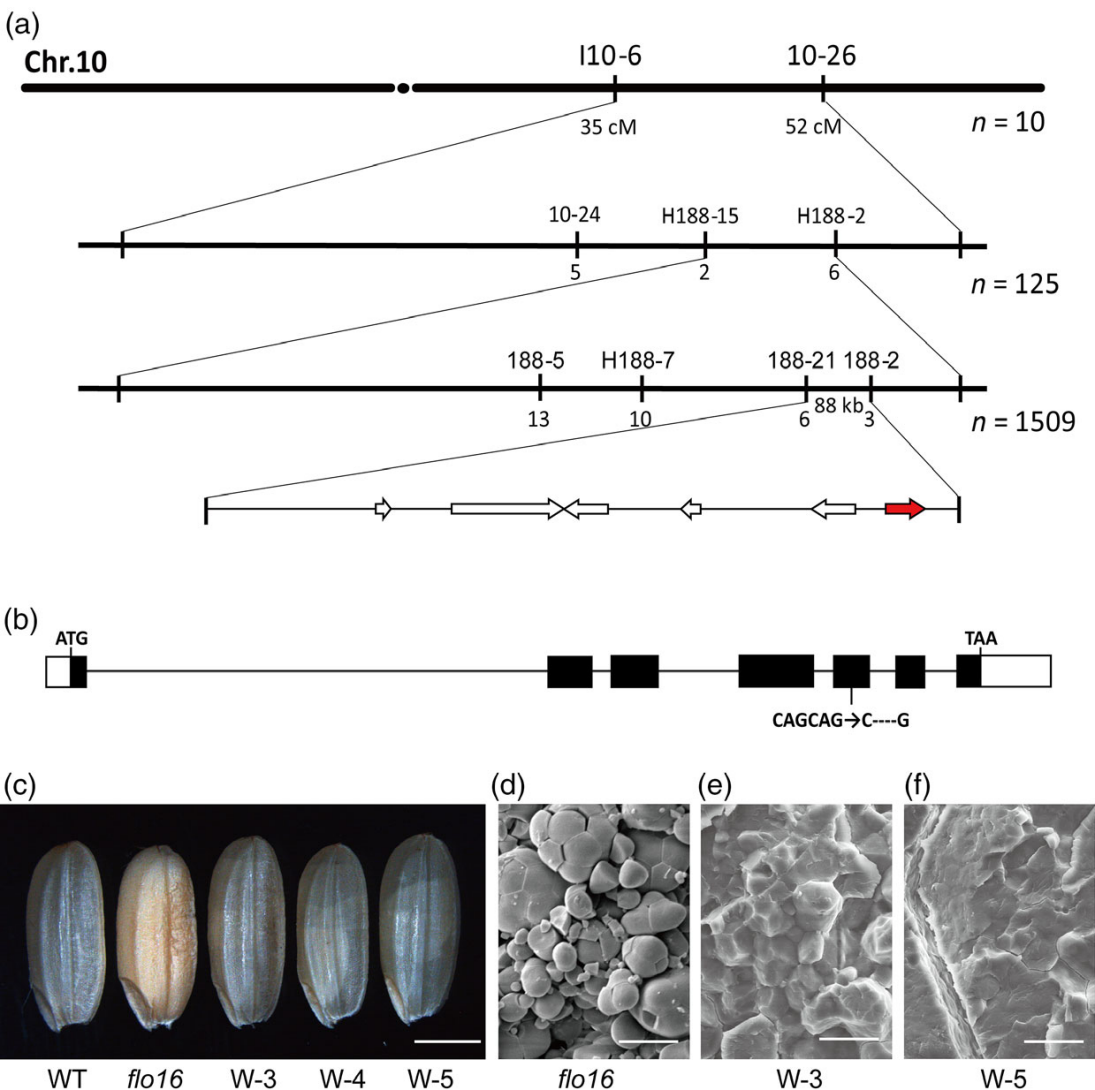
Map‐based cloning of *FLO16*. (a) Fine mapping of the *FLO16* locus. The *FLO16* locus was mapped to an 88 kb region by markers 188–21 and 188–2 on chromosome 10 (Chr.10), containing six predicted genes. Numbers of recombinants are indicated below the map. (b) The *flo16* mutant has a 4 bp deletion in the fifth exon of *Os10 g0478200*. White boxes indicate untranslated regions; black boxes indicate exons; lines indicate the introns. (c‐f) Functional complementation lines of *flo16* restore normal seed appearance. Complemented seeds became translucent (c), and SGs were restored to normal (d‐f). W‐3, W‐4 and W‐5 are representative positive transgenic lines. Scale bars, 2 mm in (c), 25 μm in (d‐f).

When *flo16* was crossed with wild type, approximately one‐quarter of the F_2_ seeds had floury endosperm (296 of 1,205, χ^2^_3:1_ = 0.122 < χ^2^_0.05,1_ = 3.84), indicating that the mutant phenotype was inherited as a single recessive allele. We then undertook map‐based cloning to identify the underlying gene. First, 10 individuals with floury endosperm selected from the F_2_ progeny of cross *flo16* (*indica*) × DJY (*japonica*) were used for linkage detection. The *FLO16* locus was initially mapped between markers I10‐6 and 10‐26 on the long arm of chromosome 10. Then, 125 *flo16* individuals were used to position *FLO16* between markers H188‐15 and H188‐2. Finally, 1,502 individuals with the recessive phenotype reduced the position of *FLO16* to an 88 kb region between markers 188–21 and 188–2 (Figure 4a; Table S3). Six predicted open reading frames (ORFs) in the region were sequenced, and we found a deletion of 4 base pairs in the coding region of *Os10 g0478200*, leading to a frame shift from amino acid residue 229 (Figure 4b), and a premature termination codon. *Os10 g0478200* was predicted to encode a cytosolic malate dehydrogenase (CMDH) composed of 332 amino acids.

To verify the identity of *flo16*, we cloned the genomic sequence of *FLO16* starting from 2,000 nucleotides upstream of the putative starting codon (ATG) to the stop codon (TAA) of *Os10 g0478200* into a plant expression vector, which was then introduced into *flo16*. Seed and SG morphologies of positive transgenic plants were similar to wild‐type plants, indicating successful rescue of the defects caused by the *flo16* mutation (Figure 4c‐f). Therefore, *Os10 g0478200* was confirmed to be the gene responsible. In addition, we generated transgenic lines expressing *CMDH* driven by *Glutelin C* promoter in *flo16*, and the endosperm‐specific expression of *CMDH* rescued the phenotype of the mutant (Figure S1), indicating that the mutant phenotype in *flo16* was mainly due to the defects in seeds rather than in vegetative tissues.

### FLO16 is cytosol‐located protein that is ubiquitously expressed in rice

**Figure 5:**
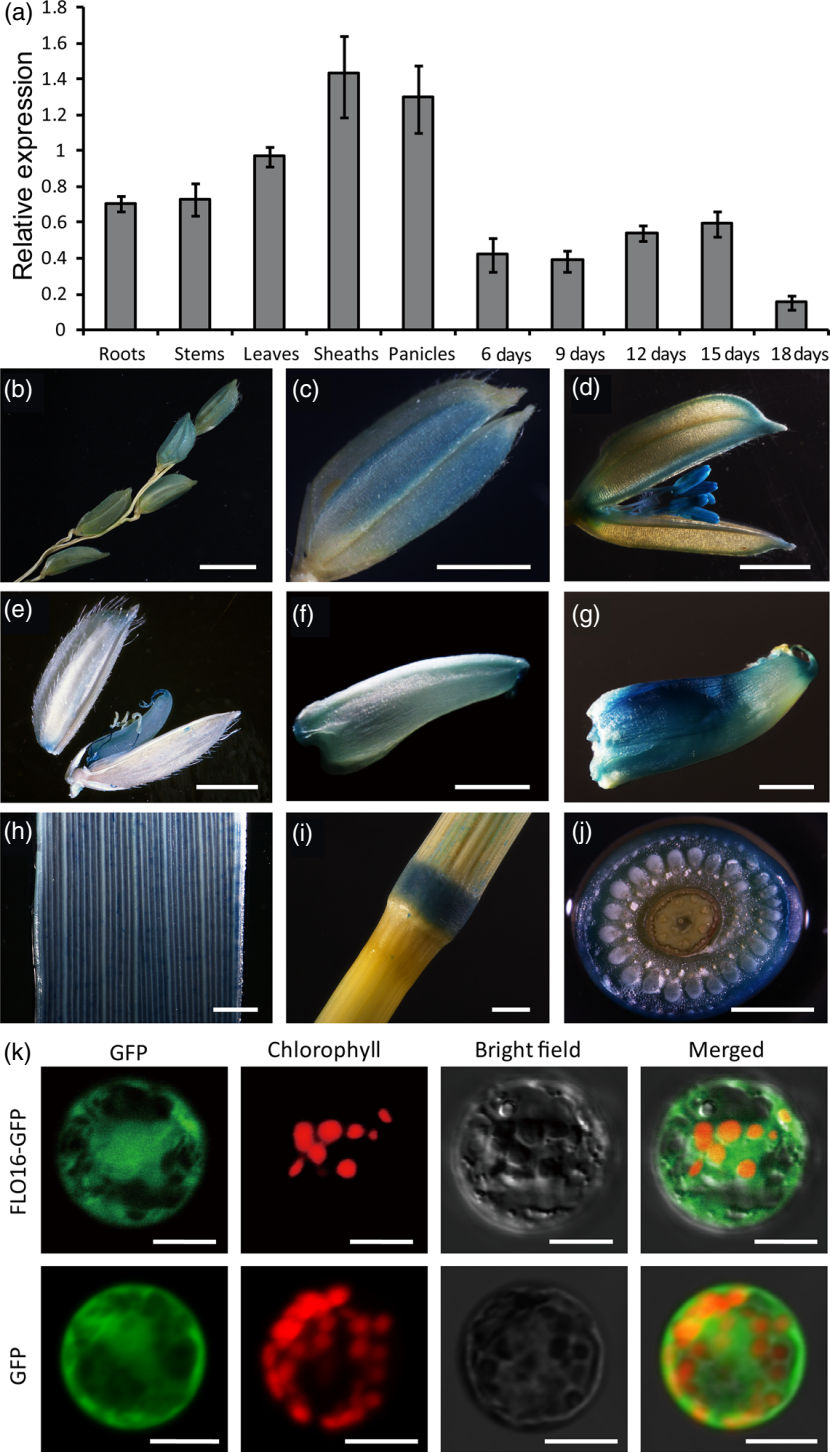
Spatial expression patterns of *FLO16*. (a) Expression levels of *FLO16* in various tissues. Developing seeds were sampled at 6, 9, 12, 15 and 18 days after flowering (DAF), and other tissues were sampled at heading. The value of *Actin I* mRNA was used as an internal control for data normalization. Values are means ± SD,* n *= 3. (b‐g) GUS activity in panicles (b), spikelet before heading (c), during heading (d), and at 3 DAF, and grains at 3 (e), 6 DAF (f) and 9 DAF (g), leaf (h), node (i, j). (j) Cross section of the node in (i). Scale bars, 10 mm in (b), 2 mm in (c‐j). (k) Subcellular localization of FLO16. Free GFP served as a control (lower panel). The fusion construct *FLO16‐GFP* was expressed in the rice protoplasts (upper panel). Green fluorescence signals, red chlorophyll autofluorescence, and bright field and merged images are shown in each panel. Scale bars, 10 μm.

We performed quantitative RT‐PCR (qRT‐PCR) analyses in wild‐type plants to investigate the expression profile of *FLO16*. Expression levels of *FLO16* in vegetative tissues, especially leaf sheaths and panicles, were higher than in the developing endosperm. Expression of *FLO16* during endosperm development increased at 6–15 DAF, but reduced at 18 DAF (Figure 5a). Hence, *FLO16* expression occurs in the early and mid‐endosperm development stages, corresponding with the time of starch biosynthesis. GUS staining in transgenic lines carrying a β*‐glucuronidase* (*GUS*) expression vector driven by the *FLO16* promoter confirmed that *FLO16* was ubiquitously expressed in rice. *FLO16* also showed a relatively high level of expression in anthers, early developing grains and stem nodes, and had a spotty distribution in leaves (Figure 5b‐j). Constitutive expression suggested that functions of *FLO16* might not be limited to the endosperm.

The *FLO16* gene was predicted to encode a cytosolic protein. To verify the subcellular location, the coding sequence of *FLO16* was C‐terminally fused with *GFP* driven by the cauliflower mosaic virus 35S (CaMV35S) promoter. Free GFP in the control was diffused evenly in cytoplasm (Figure 5k lower panel). The distribution pattern of the FLO16‐GFP signal (Figure 5k upper panel) was almost the same as that of free GFP, suggesting that FLO16 protein was diffused in the cytosol as predicted.

### Disruption of FLO16 causes multiple metabolic changes

**Figure 6:**
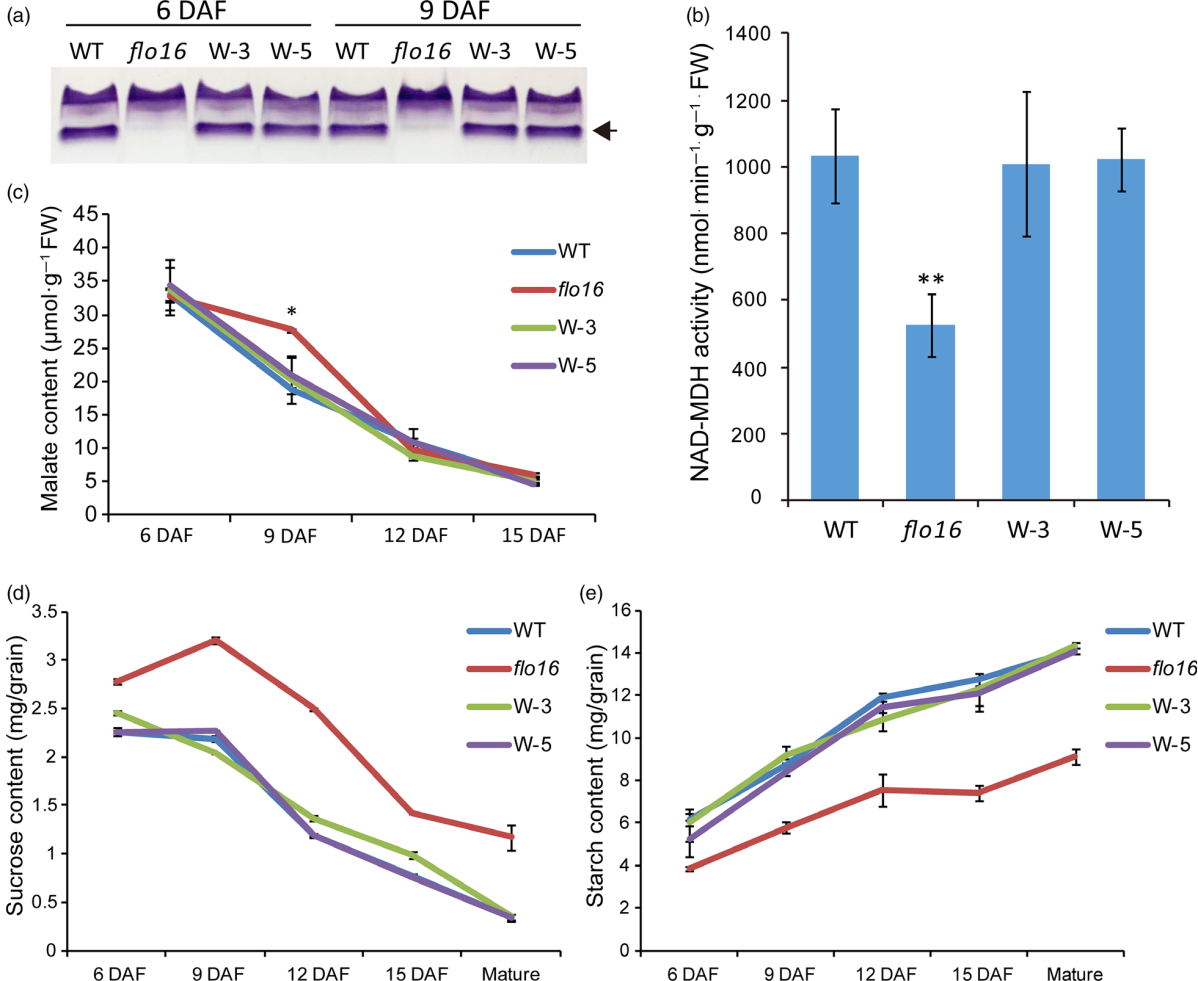
Responses of the metabolic pathways to deletion of FLO16 activity. (a) Native PAGE profiles of malate dehydrogenase (MDH) activities in developing endosperms at 6 and 9 DAF. (b) Malate contents in developing endosperms. (c) NAD‐MDH activity in wild‐type and *flo16* developing endosperm. (d) Sucrose contents in developing and mature endosperm. (e) Starch contents in wild‐type and *flo16* endosperm. All values are means ± SD,* n *= 3. Asterisks indicate statistical significance between the wild type and mutant, determined by Student's *t*‐tests (*P < 0.05; **P < 0.01).

MDH isoforms catalyse the reversible conversion of malate to OAA (Gietl, 1992; Heber, 1974; Scheibe, 2004). To determine endogenous MDH activity, crude enzyme was extracted from developing endosperm 6 and 9 DAF for native polyacrylamide gel electrophoresis (PAGE) analysis. As expected, one of the activity bands was not detected in the *flo16* mutant, which was recovered in the complementation lines. Thus, *flo16* seems to be a knockout mutant of CMDH. Interestingly, other activity bands showed a slight compensatory increase (Figure 6a). The total NAD‐MDH activity in *flo16* endosperm at 9 DAF was less than one‐half of that in the wild type (Figure 6b). The lack of CMDH activity might result in alterations in substrates and products of malate metabolism. We evaluated the levels of closely related metabolites. There was an increase in malate levels in young leaves and roots relative to wild type, and malate levels in grains were increased only at 9 DAF (Figures S3A and 6c). We inferred that malate was mainly the substrate of CMDH. Considering that a ‘malate valve’ plays an essential role in redox regulation and energy conversion (Scheibe, 2004), we evaluated the major form of redox power and energy supplying, and identified decreases in the NADPH and ATP content (Figure S2). This indicated that energy metabolism and redox homeostasis were unstable in the *flo16* mutant.

### Defective starch biosynthesis in *flo16*

**Figure 7:**
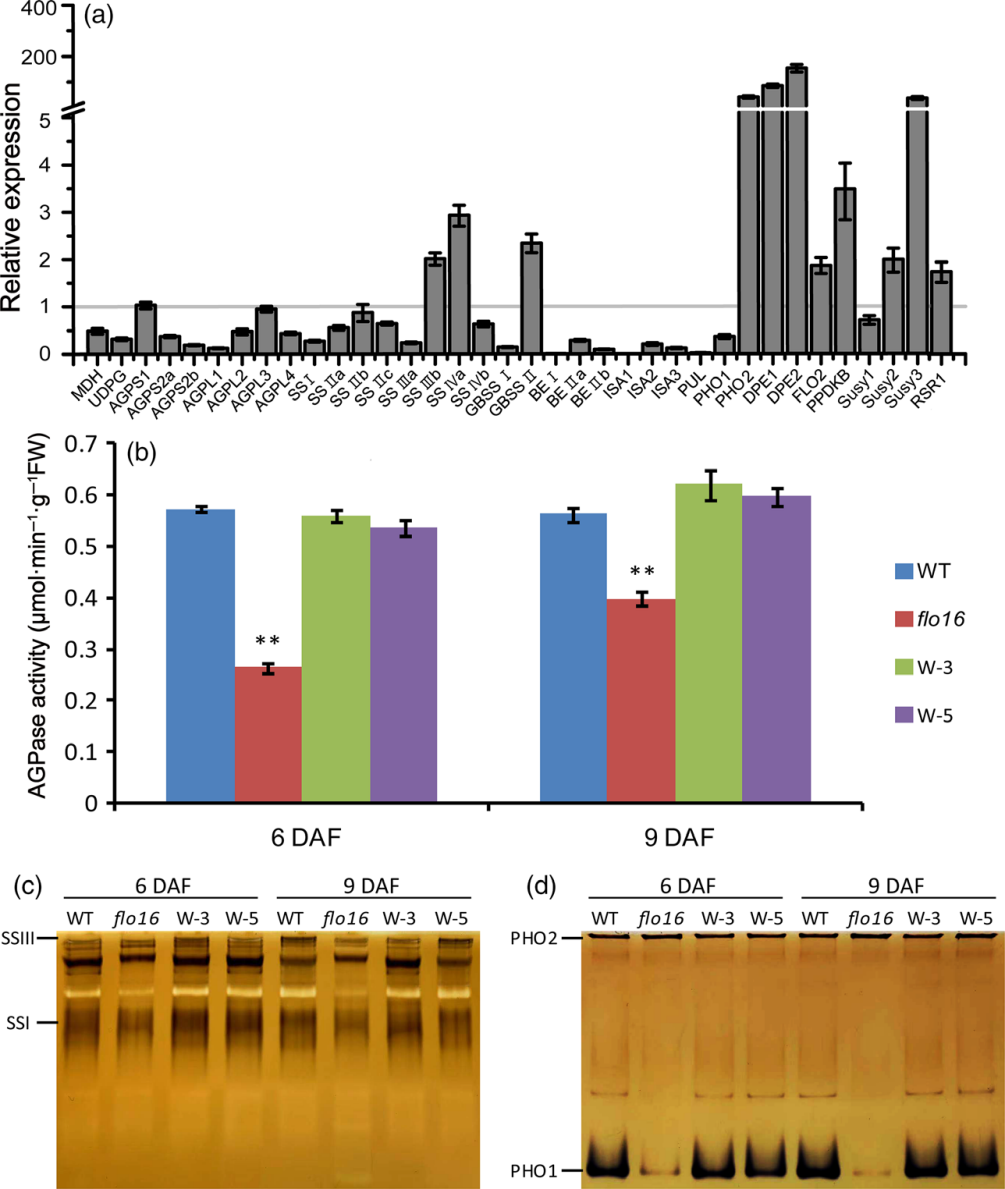
Gene expression and protein activity analyses of starch synthesizing enzymes. (a) Expression levels of starch synthesis‐related enzymes in developing endosperm 10 DAF. Data represent ratios of expression levels in *flo16* to that of wild type. Values are means ± SD,* n *= 3. (b) AGPase activities in developing endosperm of wild type and *flo16*. Values are means ± SD,* n *= 3. Asterisks indicate the statistical significance determined by Student's *t*‐tests (*P < 0.05; **P < 0.01). (c) Activity bands for SSI, and SSIII. (d) Activity bands for PHO1 and PHO2.

Sucrose contents in single developing and mature grains of *flo16* were much higher than in the wild type (Figure 6d), while starch contents were obviously lower (Figure 6e). Real‐time quantitative RT‐PCR was performed to examine possible effects of FLO16 on starch biosynthesis. Expression levels of many genes participating in starch biosynthesis during endosperm development were decreased, including genes coding for UDPG pyrophosphatase (UGPase), AGPases (AGPL2 and AGPS2b), soluble SSs (SSI), GBSSI, BEI, BEIIb, isoamylases (ISA1 and ISA2) and pullulanase (PUL). In contrast, genes coding for cytosolic phosphorylase (PHO2), disproportionating enzyme (DPE1 and DPE2) and sucrose synthase (Susy3) were increased, whereas *FLO2*,* PPDKB* (*Pyruvate orthophosphate dikinase B*) and *RSR1* (*Rice starch regulator 1*) were only slightly increased (Figure 7a). It seems that genes functioning upstream of *AGPase* were highly expressed, and those acting downstream of *AGPase* were significantly reduced.

The activities of key enzymes in starch synthesis were investigated. We first investigated AGPase activity since it was the rate‐limiting enzyme in starch synthesis. As predicted, AGPase activity in developing *flo16* endosperm was lower than in the wild type (Figure 7b). Zymogram analyses performed to detect activities of several essential starch synthetic enzymes showed decreased activities of SSI (Figure 7c) and PHO1 (Figure 7d) in the *flo16* mutant relative to wild type, but there was no significant change in SSIII and PHO2 activity. Thus, mutation of the *FLO16* gene showed obvious effects on the activities of the key starch synthetic enzymes.

### Starch biosynthesis in *flo16* has almost no respond to exogenous malate or sucrose

**Figure 8:**
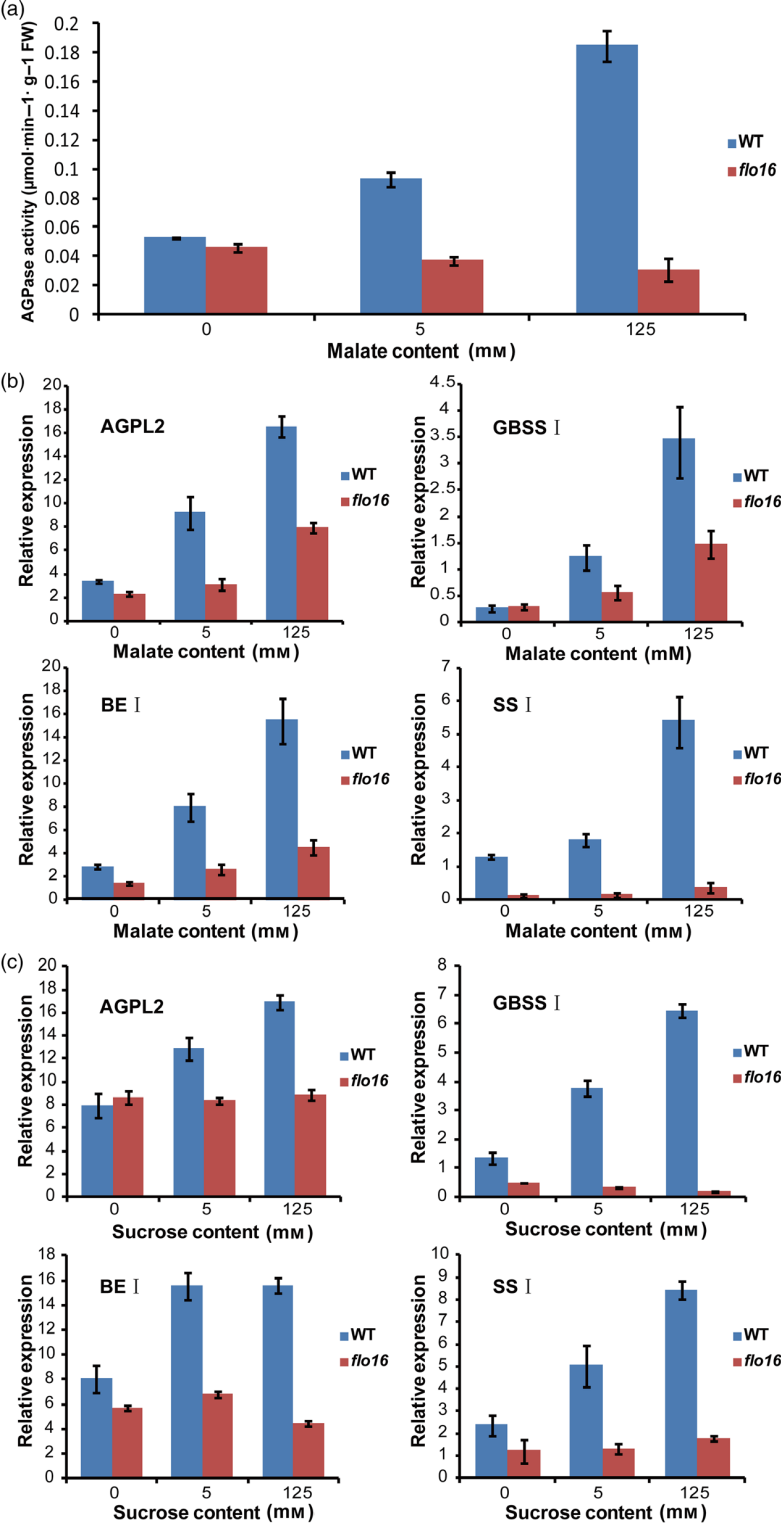
Influence of exogenous malate and sucrose on starch synthesis enzymes in wild‐type and *flo16* endosperm. (a) AGPase activities in wild‐type and *flo16* developing endosperms at 9–12 DAF treated with malate. (b) Expression levels of starch synthesis enzymes in the developing endosperm at 9–12 DAF incubated in malate. (c) The Expression levels of starch synthesis enzymes in developing endosperm at 9–12 DAF treated with sucrose. Values are means ± SD,* n *= 3.

Alterations in malate level caused dramatic effects on transitory starch metabolism in tomato fruit (Centeno *et al*., 2011). However, no difference in starch content was observed when malate level was decreased in potato (Szecowka *et al*., 2012). We investigated the influence of malate on starch synthesis in the developing endosperm of rice. Isolated *flo16* and wild‐type endosperms at 9–12 DAF were incubated in the presence of 0, 5 or 125 mM malate for 3 h, respectively. Endosperm discs were washed twice, and the activities of starch synthesis‐related enzymes were determined. Catalytic activity of AGPase in the wild type was induced by malate treatment, whereas that in *flo16* was significantly lower and barely responded to malate treatment (Figure 8a). Concurrently, expression levels of several key enzymes in starch synthesis were tested for induction in wild‐type endosperm (Figure 8b). Those experiments revealed up‐regulation in starch biosynthetic flux, suggesting that exogenous malate promoted transition from sucrose to starch. The deletion of CMDH at least partly blocked the response of starch synthesis to malate. Similar results were obtained when developing endosperms of *flo16* and wild type were treated with sucrose (Figure 8c). Thus, the starch synthesis in *flo16* mutant is defective in response to exogenous malate and sucrose.

### Overexpression of *FLO16* increases grain weight

**Figure 9:**
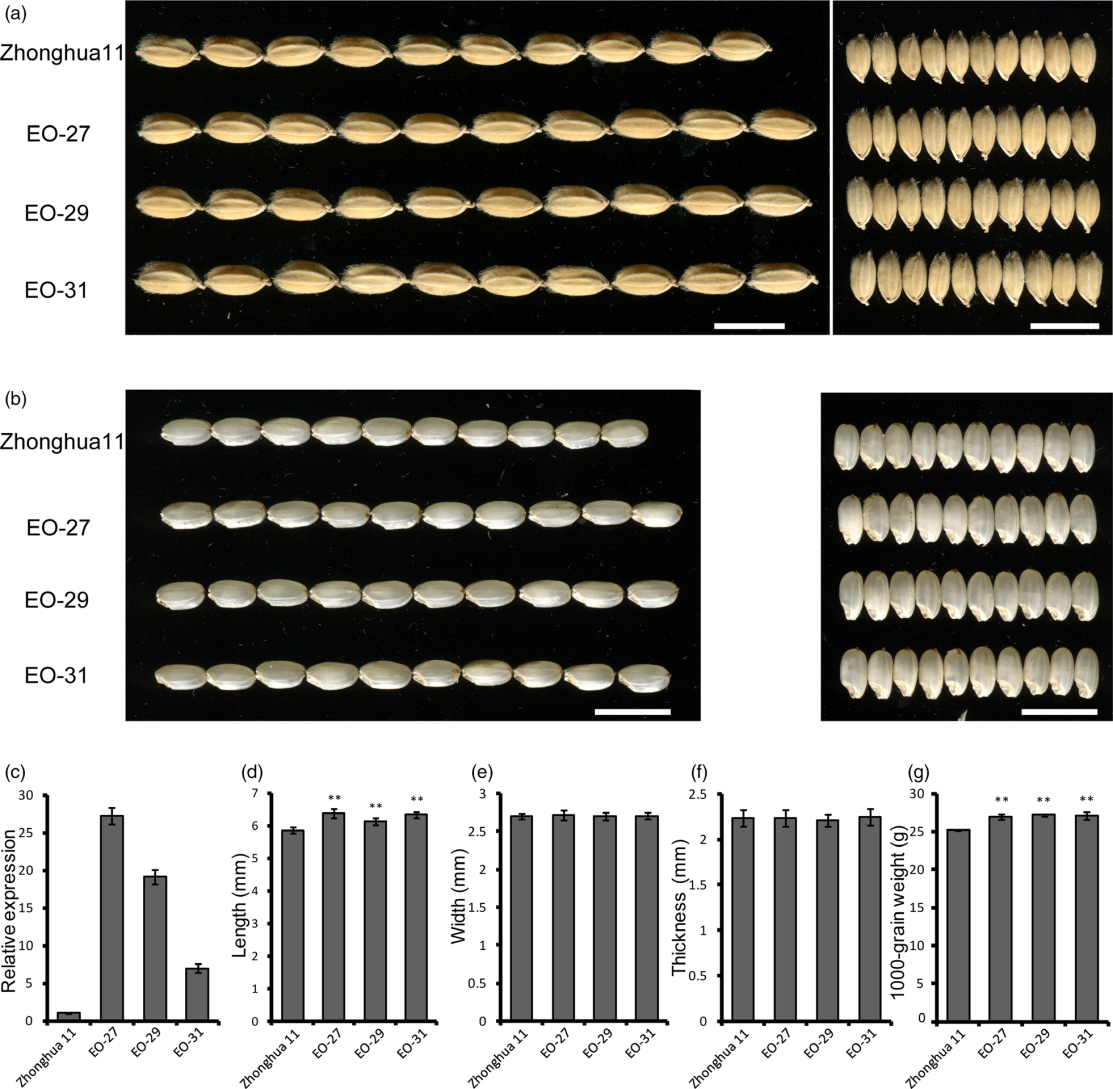
Effects of endosperm‐specific *FLO16* overexpression on grains. (a, b) Images of hulled (a) and dehulled (b) seeds of the recipient and transgenic lines. EO‐27, EO‐29 and EO‐31 are independent transgenic lines. Scale bars, 1 cm. (c) Enhanced expression levels of *FLO16* in overexpression endosperms. Expression level in Zhonghua 11 was considered as sample control. Values are means ± SDs, *n *= 3. (d–f) Size comparisons between the wild‐type and transgenic seeds. Values are means ± SDs, *n *= 10. (g) 1000‐grain weights of overexpression lines. Values are means ± SDs, *n *= 3. Asterisks indicate the statistical significance between the wild type and the mutant, determined by Student's *t*‐tests (**P < 0.01).

We created transgenic lines overexpressing *FLO16* driven by the endosperm‐specific *Glutelin C* promoter to evaluate the effect of elevated *FLO16* expression level in rice endosperm. qRT‐PCR analysis showed that the expression levels of *FLO16* in positive transgenic lines were much higher than those in the endosperm of the recipient cultivar Zhonghua 11 (Figure 9c). The major agronomic traits of these transgenic plants showed almost no difference with the recipient (Table S2). Notably, the 1000‐grain weight of these lines were significantly higher than Zhonghua 11 (Figure 9g), indicating that endosperm‐specific overexpression of *CMDH* will significantly promote starch biosynthesis in rice. Further analysis showed the elevated grain weight are mainly to the enlarged grain size as the grain length of transgenic lines were much larger than Zhonghua 11 (Figure 9a,d). This trend was even more obvious when dehulled grains were compared (Figure 9b). It is noteworthy that constitutive overexpression of *CMDH* could also result in increased grain weight (Figure S5).

## Discussion

### CMDH has strong effects on starch biosynthesis during seed development

Previous studies characterized several mutants that affected starch synthesis in rice. Many of them have opaque endosperm, such as *flo5* (*ssIIIa*), *waxy* (*gbssI*) and *amylose‐extender* (*ae*,* beIIb*) (Nishi *et al*., 2001). Endosperm‐defective mutants are valuable resources for elucidating molecular mechanisms underlying seed development and/or starch biosynthesis. In this study, we isolated mutant *flo16* with floury endosperm and retarded growth. Its opaque endosperm appearance seemed to be caused by defective SG formation (Figure 2). Further analyses revealed that reduction in starch synthesis in *flo16* endosperm led to reduced grain weight (Figure 1f), and changes in amylose content and amylopectin structure (Figure 3a,b).

The *FLO16* locus was located within an 88 kb region in chromosome 10S. Sequence analysis and subsequent complementation tests determined that the underlying gene was *Os10 g0478200*. A 4 bp deletion caused premature termination of FLO16, indicating that *flo16* is most probably a knockout mutant of that gene (Figure 4). MDH activity assays confirmed this result (Figure 6a). *Os10 g0478200* encodes a cytosolic malate dehydrogenase (CMDH) that is expressed ubiquitously. Expression of *CMDH* driven by endosperm‐specific *Glutelin C* and *Ubiquitin* promoters both rescued the opaque phenotype of mutant seeds, indicating that the grain phenotype is due to lack of CMDH in the endosperm. Therefore, CMDH is important for starch biosynthesis during seed development.

### CMDH functions in supplying reducing power and energy for starch biosynthesis in rice endosperm

Plant MDHs function in equilibrating reducing equivalents between organelles in the cell, and catalyse reversible conversion of malate and NAD(P) to OAA and NAD(P)H. In the cytosol, malate is oxidized to oxaloacetate by the cytosolic MDHs, generating reducing equivalent in the form of NADH. Oxaloacetate is then transported to the plastids, where the plastidial MDH converts it back to malate. This so‐called ‘malate valve’ is vital in equilibrating reducing equivalents in the cytosol and between organelles as well as regulating malate for further metabolic synthesis (Scheibe, 2004; Taniguchi and Miyake, 2012). An *Arabidopsis* double mutant lacking both the mitochondrial MDH isoforms was defective in seed maturation and post‐germination growth (Sew *et al*., 2016; Tomaz *et al*., 2010), while the mutant deficient in peroxisomal isoforms requires exogenous sugars for seedling (Pracharoenwattana *et al*., 2010). Plastidial NADP‐MDH can be redox‐regulated and activated dependent on light (Scheibe, 1987), while plastidial NAD‐MDH is not redox sensitive and acts in dark and non‐green parts (Berkemeyer *et al*., 1998). Plant growth of *Arabidopsis nadp‐mdh* mutants was either not affected (Hebbelmann *et al*., 2012) or slight weakened (Heyno *et al*., 2014) compared to the wild type, while *pdnad‐mdh* knockout mutant has an embryo–lethal phenotype (Beeler *et al*., 2014). Although it is well established that MDHs play important roles in central metabolism in plants, no *mdh* mutants were reported in rice. Recently, Heng *et al*. (2018) revealed that the defect in malate transport results in apical abortion panicles, suggesting an important role of malate metabolism in rice. However, the importance of CMDH in rice remains unclear.

In this study, CMDH activity was not detected in the *flo16* mutant, resulting in overaccumulated malate and decreased NADPH and ATP contents (Figures 6c and S2). This result suggested that rice CMDH mainly functions in converting malate and NAD(P) to OAA and NAD(P)H. Further exogenous malate treatment indicated that high levels of malate had no detrimental effect on starch biosynthesis (Figure 8a,b). Sucrose flux is the main form of substrate supply for starch synthesis in the endosperm. Exogenous sucrose in sweet potato increased the transcript level of *GBSSI* (Wang *et al*., 2001). In our study, expression levels of *GBSSI* and several key enzymes in the wild type were induced by sucrose and malate, whereas no significant differences were observed in *flo16* (Figure 8), indicating that starch synthesis in response to malate and sucrose is blocked in the *flo16* mutant. Starch synthesis requires a high rate of turnover of reducing equivalents and a mass of energy supply. Previous study in tomato showed that alterations in mitochondrial malate metabolism have strong effects on starch biosynthesis in the amyloplast due to an altered cellular redox status (Centeno *et al*., 2011). In *flo16*, significantly decreased NADPH content indicated a lack of reduction potential required for starch synthesis. Moreover, ATP is referred to as the energy currency of the cell. Lower level of ATP in *flo16* is expected to result in a shortage of energy, which in turn greatly affects starch biosynthesis. Therefore, it is likely that alterations in cytosolic malate‐related metabolism have strong effects on starch biosynthesis during seed development. Notably, mutation of CMDH led to defective plant growth, increased malate and reduced ATP content in *flo16* seedling, indicating that the malate‐related metabolism also existed in other part of rice plant (Figures 1c,d and S3). However, the endosperm‐specific expression of *CMDH* almost completely rescued these deficiencies. Considering the significant role of photosynthesis in regulating redox state, we thus speculated that CMDH is more important in sink organs than source organs in rice.

Previous study in potato declared that the redox state affects starch synthesis by post‐translational regulation of AGPase (Tiessen *et al*., 2002). Moreover, the redox control of AGPase activity was also reported in rice endosperm (Tuncel *et al*., 2014). However, our study clearly indicates that AGPase activity is independent of redox control as the decrease ratio was constant (Figure S4). Whether and how reducing equivalents alter the redox state of other starch synthesis‐related enzymes remains to be further addressed.

### Overexpression of *FLO16* increases grain weight in rice

Grain weight is the major component of crop yield and an important agronomic trait in the breeding of cereal crops. Grain weight is positively associated with grain size. Several genes affecting grain weight have been identified in rice, such as *GS3* (Fan *et al*., 2006; Mao *et al*., 2010), *GW2* (Song *et al*., 2007), *qSW5*/*GW5* (Liu *et al*., 2017; Shomura *et al*., 2008; Weng *et al*., 2008), *GS5* (Li *et al*., 2011), *qGL3/qGL3.1* (Qi *et al*., 2012; Zhang *et al*., 2012), *GW8* (Wang *et al*., 2012), *TGW6* (Ishimaru *et al*., 2013),*GL7/GW7* (Wang *et al*., 2015a,b), *Gn1a* (Ashikari *et al*., 2005), *DEP1* (Huang *et al*., 2009) and *OsSPL14* (Jiao *et al*., 2010), but the mechanisms that control grain weight are rarely reported.

Our endosperm‐specific overexpressing of *CMDH* in Zhonghua 11 significantly increased grain weight which might due to the elevated grain length, demonstrated that elevated CMDH activity in the endosperm significantly promotes starch biosynthesis. Therefore, FLO16 has the potential for improving grain weight and grain yield in rice.

## Experimental procedures

### Plant materials and growing conditions

The *flo16* mutant was generated by ^60^Co treatment of *indica* cv. N22 (Nagina22, an Indian traditional variety). An F_2_ population from cross *flo16* mutant × *japonica* cv. DJY (Dianjingyou 1, from Yunnan Academy of Agricultural Sciences, *Japonica* Rice Breeding Center) was developed for fine mapping. Rice plants were grown in experimental fields at Nanjing or Beijing. Developing seeds of the wild type (N22) and *flo16* at 6–21 days after flowering (DAF) were harvested and stored at −80°C if not used immediately.

### Starch grain observation

Transverse sections (~1 mm in thickness) of developing endosperm were fixed in 2% (W/V) paraformaldehyde, 2% (V/V) glutaraldehyde and 250 mm sucrose buffered with 50 mm PIPES‐KOH (pH 7.2). The fixed endosperm was then dehydrated in an ethanol series and embedded in LR White resin (London Resin, Berkshire, UK). After sectioning with an ultramicrotome (UC7, Leica, Solms, Germany), specimens (1 μm) were stained with I_2_‐KI and observed under an optical microscope (Nikon, Tokyo, Japan).

Naturally fractured sections of mature seeds were observed with a scanning electron microscope (S‐3400N, Hitachi, Tokyo, Japan) as described (Kang *et al*., 2005).

### Physicochemical properties of starch in endosperm

Starch content in the endosperm was measured using a starch assay kit (Megazyme, Ireland). Amylose, lipid and protein contents were measured as described previously (Kang *et al*., 2005; Liu *et al*., 2009). Chain length distribution of amylopectin was measured using DSA‐FACE (Han *et al*., 2012). Pasting properties were determined with a rapid visco analyzer (RVA‐Tec Master, Perten, Sweden). Gelatinization and swelling coefficient of starch were measured by mixing flour with different concentrations of urea (0–9 m) and incubating at 25°C for 24 h (Nishi *et al*., 2001).

### Mapping and cloning of *FLO16*

A total of 1502 F_2_ individuals with recessive phenotype were used for map‐based cloning. Molecular markers were developed according to nucleotide polymorphisms between N22 and DJY (Table S3). Six open reading frames (ORFs) in the fine‐mapped region were predicted by the Rice Annotation Project Database (http://rapdb.dna.affrc.go.jp/). The mutation was confirmed by sequencing PCR products for these genes.

### Vector construction and plant transformation

For functional complementation of *flo16*, the wild‐type *FLO16* sequence under control of its native promoter (2000 bp upstream of ATG) was inserted into vector pCUbi1390. This plasmid was introduced into *flo16* callus by Agrobacterium‐mediated transformation (Hiei and Komari, 2008; Hiei *et al*., 1994). For endosperm‐specific functional complementation of *flo16* and overexpression of *FLO16*, the wild‐type *FLO16* sequence was inserted into vector pCUbi1390, in which *FLO16* was driven by the *Glutelin C* promoter. This plasmid was introduced into *flo16* and Zhonghua 11 callus, respectively. For constitutive overexpression of *FLO16*, the wild‐type *FLO16* sequence was inserted into vector pCUbi1390, in which *FLO16* was driven by the maize *Ubiquitin* promoter, and this plasmid was introduced into Zhonghua 11 callus. For promoter analysis, the *FLO16* promoter was inserted into a β*‐glucuronidase* (*GUS*) expression vector and introduced into Nipponbare callus as described above. All primers used were listed in Table S4.

### GUS staining

Transgenic plants obtained as mentioned above were stained as described (Jefferson *et al*., 1987). Images were captured with a stereoscope (Leica Application Suite 3.3, Germany)

### Subcellular localization

The coding sequence of *FLO16* was cloned into the pAN580‐GFP vector to express *FLO16‐GFP* under the control of a double 35S promoter. The control (*GFP* alone) and *FLO16‐GFP* were each expressed in rice protoplasts (Chen *et al*., 2006). Images of GFP fluorescence were captured using a confocal laser scanning microscope (LSM710, Zeiss, Germany).

### RNA extraction and qRT‐PCR analysis

Total RNA was extracted using an RNA Prep Pure Plant kit (TIANGEN Biotech, Beijing). First‐strand cDNA was synthesized from 2 μg of total RNA by priming with oligo (dT) in 20 μL reaction volumes, using a PrimeScript Reverse Transcriptase Kit (TaKaRa, Tokyo, Japan). qRT‐PCR was performed using the SYBR Premix Ex Taq (TaKaRa) in an ABI7500 Real‐time PCR system. Gene‐specific primers used in this analysis are listed in Table S3 or the previous study (She *et al*., 2010). *ActinI* was used as an internal control.

### Determination of metabolite levels

Levels of sucrose were quantified by UPLC as described previously (Fernie *et al*., 2001). Malate contents were measured by enzymatic analysis (Nunes‐Nesi *et al*., 2007). ATP contents were detected using an ATP assay kit (Beyotime, Shanghai, China) according to the manufacturer's instructions. Developing grains and other tissues of rice were captured into liquid N_2_ and stored in −80°C if not extracted immediately, and all extractions were performed on ice as soon as possible to reduce loss of substance. Furthermore, the extractions for ATP and malate measurement were filtered across the 10 kDa ultrafiltration centrifugal tube (Millipore, Billerica, MA) to decrease degradation of the metabolites.

### Enzyme activity and zymogram analysis

Crude enzymes from developing endosperm were extracted for enzyme activity assays (Peng *et al*., 2014). MDH isozymes were separated in 10% native polyacrylamide gels stained according to a previous study (Brown *et al*., 1978). Total MDH activity assays were performed in reaction mixtures containing 10 μL crude enzyme, 3 mm malate, 1 mm NAD buffered with 50 mm glycine‐NaOH (pH 8.5) at 25°C and determined by monitoring altered NADH content at 340 nm (Zheng *et al*., 2005). AGPase was extracted in 50 mm Hepes‐KOH (pH 7.8) buffer with 5 mm MgCl_2_ and analysed in 50 mm Hepes‐KOH (pH 7.8), 5 mm MgCl_2_, 0.6 mm NAD, 2.5 mm Na‐PPi, 1 unit/mL phosphoglucomutase (Sigma‐Aldrich, Saint Louis, MO), 2.5 units/mL Glc‐6‐P dehydrogenase (Sigma‐Aldrich) and 1 mm ADP‐Glc with or without 5 mm DTT (Tiessen *et al*., 2002). Activities measured with or without DTT were termed Vred or Vsel, respectively. Activities of starch synthases (SSI and SSIII) were determined according to Nishi *et al*. (2001). Phosphorylase (PHO1 and PHO2) activities were tested in a polyacrylamide gel containing 0.8% (w/v) oyster glycogen (Sigma‐Aldrich) (Satoh *et al*., 2008). All electrophoreses were conducted at 4°C.

### Incubation of endosperm discs with exogenous malate or sucrose

Developing seeds at 9–12 DAF were harvested and then sliced into 1‐mm‐thickness discs, washed three times in fresh incubation medium (50 mM HEPES‐KOH, pH7.4), and incubated (~200 mg) in 5 mL of incubation medium containing Na‐malate or sucrose in various concentration for 3 h. All incubations were performed at 30°C with 90 rpm shaking. After incubation, samples were washed three times in incubation medium and blotted with filter papers. Further assays were performed as mentioned above.

## Supporting information


**Figure S1** Endosperm‐specific functional complementation lines of *flo16* restore normal appearance.
**Figure S2** NADP^+^/NADPH and ATP contents in developing endosperm.
**Figure S3** Metabolic differences between wild type and *flo16* in young seedling.
**Figure S4** Redox activation state of AGPase.
**Figure S5** Effects of *FLO16* over‐expression on grains.
**Table S1** Comparison of agronomic traits between the wild type and *flo16* mutant.
**Table S2** Agronomic traits of overexpression lines.
**Table S3** Oligonucleotide primers used in map‐based cloning.
**Table S4** Gene‐specific primers used in this study.
