# Supplementary material for: FLOURY ENDOSPERM16 encoding a NAD‐dependent cytosolic malate dehydrogenase plays an important role in starch synthesis and seed development in rice
Source: Plant Biotechnol J. 2019 Mar 27;17(10):1914–27. doi: 10.1111/pbi.13108 (PMC6737025; doi:10.1111/pbi.13108)
Supplement: Supplementary file 1 — Figure S1 Endosperm‐specific functional complementation lines of flo16 restore normal appearance. Figure S2 NADP+/NADPH and ATP contents in developing endosperm. Figure S3 Metabolic differences between wild type and flo16 in young seedling. Figure S4 Redox activation state of AGPase. Figure S5 Effects of FLO16 over‐expression on grains. Table S1 Comparison of agronomic traits between the wild type and flo16 mutant. Table S2 Agronomic traits of overexpression lines. Table S3 Oligonucleotide primers used in map‐based cloning. Table S4 Gene‐specific primers used in this study. [file PBI-17-1914-s001.docx]

**Supporting Information**

**
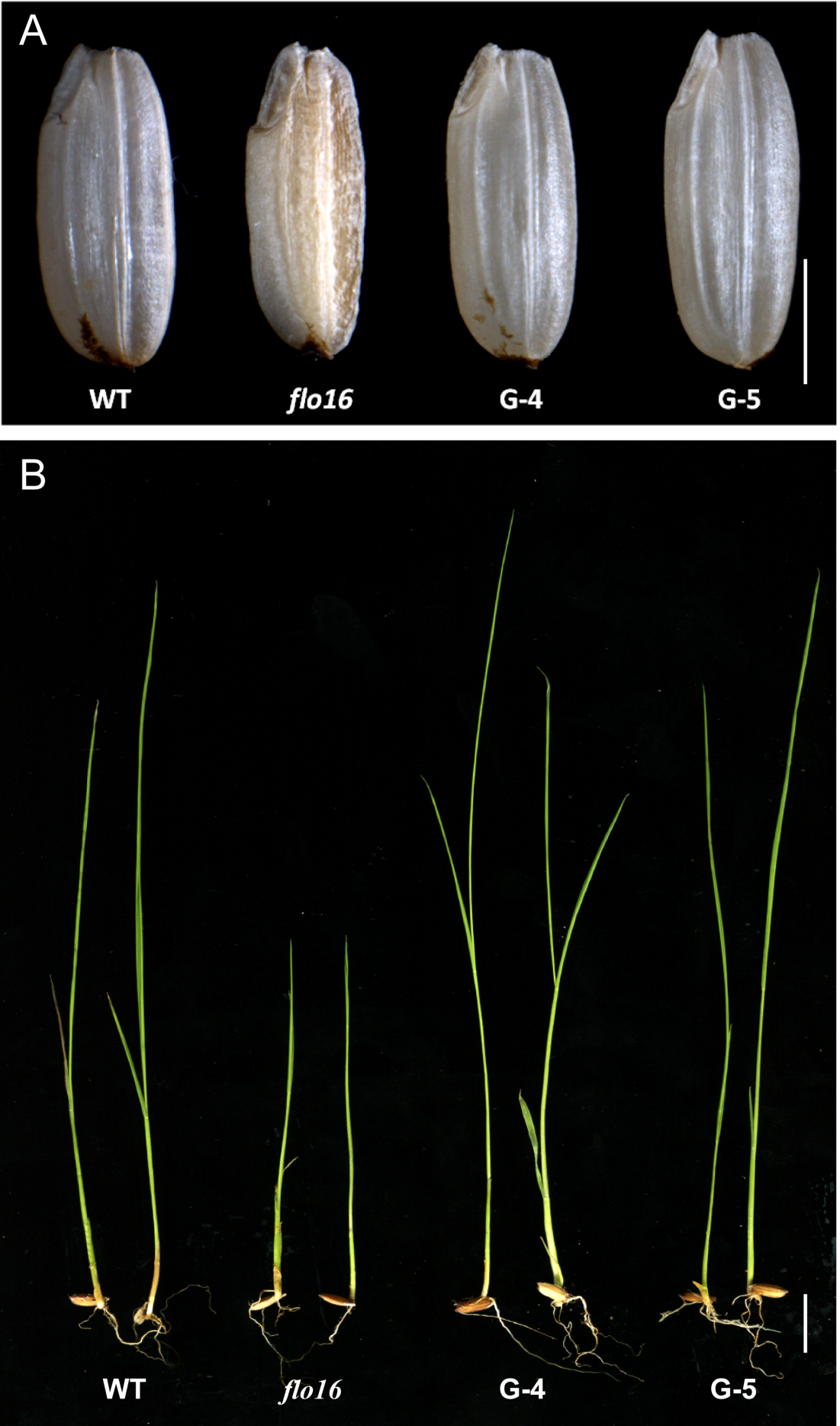
**

**Figure S1.** Endosperm-specific functional complementation lines of *flo16* restore normal appearance. G-4 and G-5 represent transgenic lines expressing *CMDH* by the grain-specific *glutelin C* promoter in *flo16*. Scale bar, 2 mm in A and 1 cm in B.

**
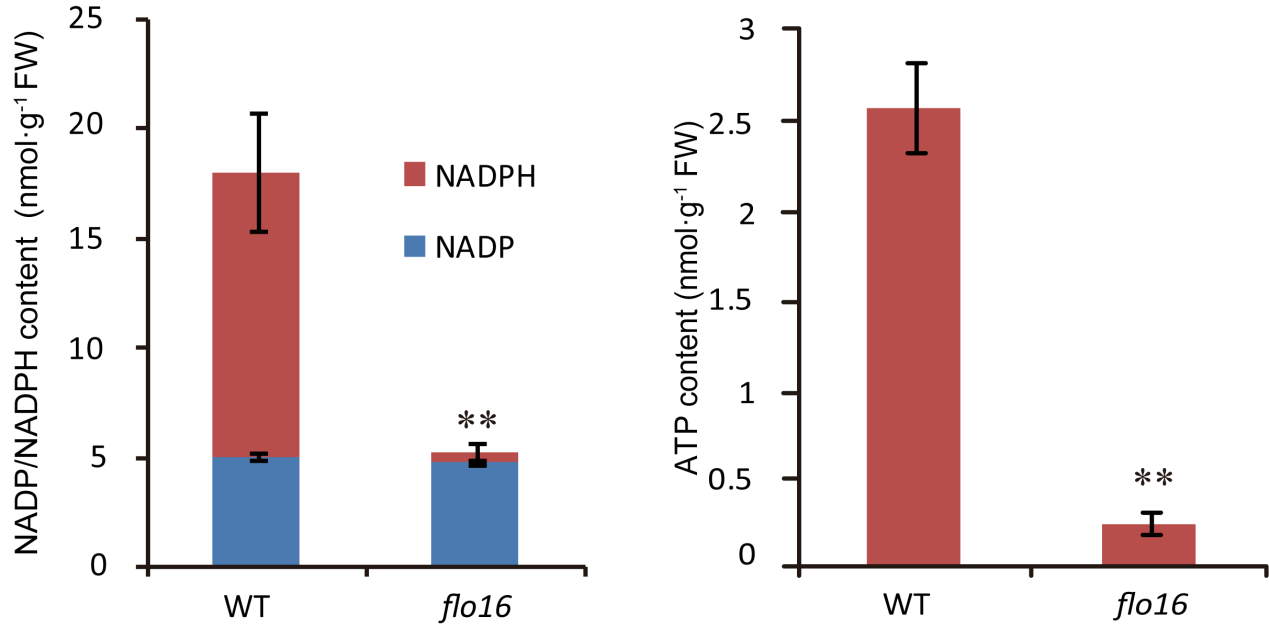
**

**Figure S2.** ATP and NADP^+^/NADPH contents in developing endosperm. Values are means ± SD, n = 3. Asterisks indicate the statistical significance between wild type and the mutant as determined by a Student’s *t*-test (*P <0.05; **P <0.01)


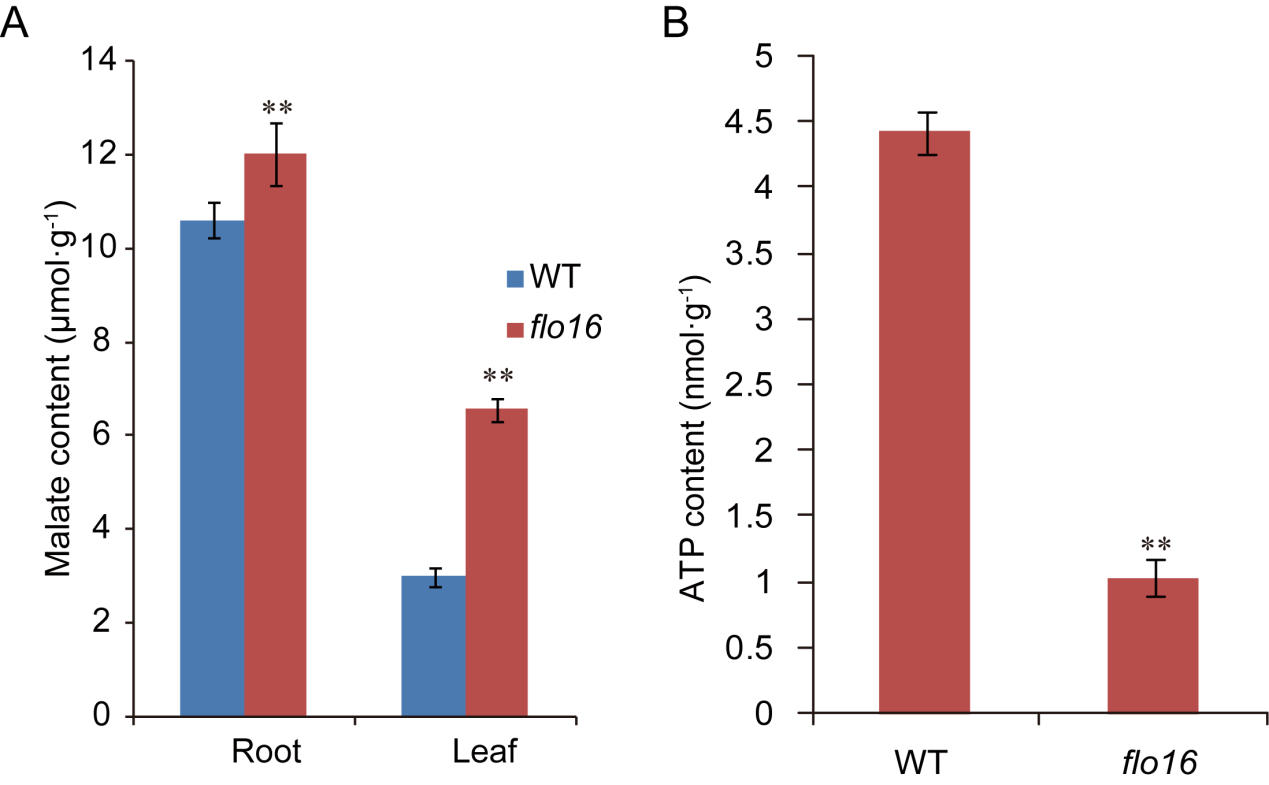
**Figure S3.** Metabolic differences between wild type and *flo16* in young seedling. (A) Malate contents in one-week-old plants of wild type and *flo16* mutant. (B) ATP contents in wild-type and *flo16* one-week-old plants. All values are means ± SD, n = 3. Asterisks indicate statistical significance between the wild type and mutant, determined by Student’s *t*-tests (**P <0.01).

*
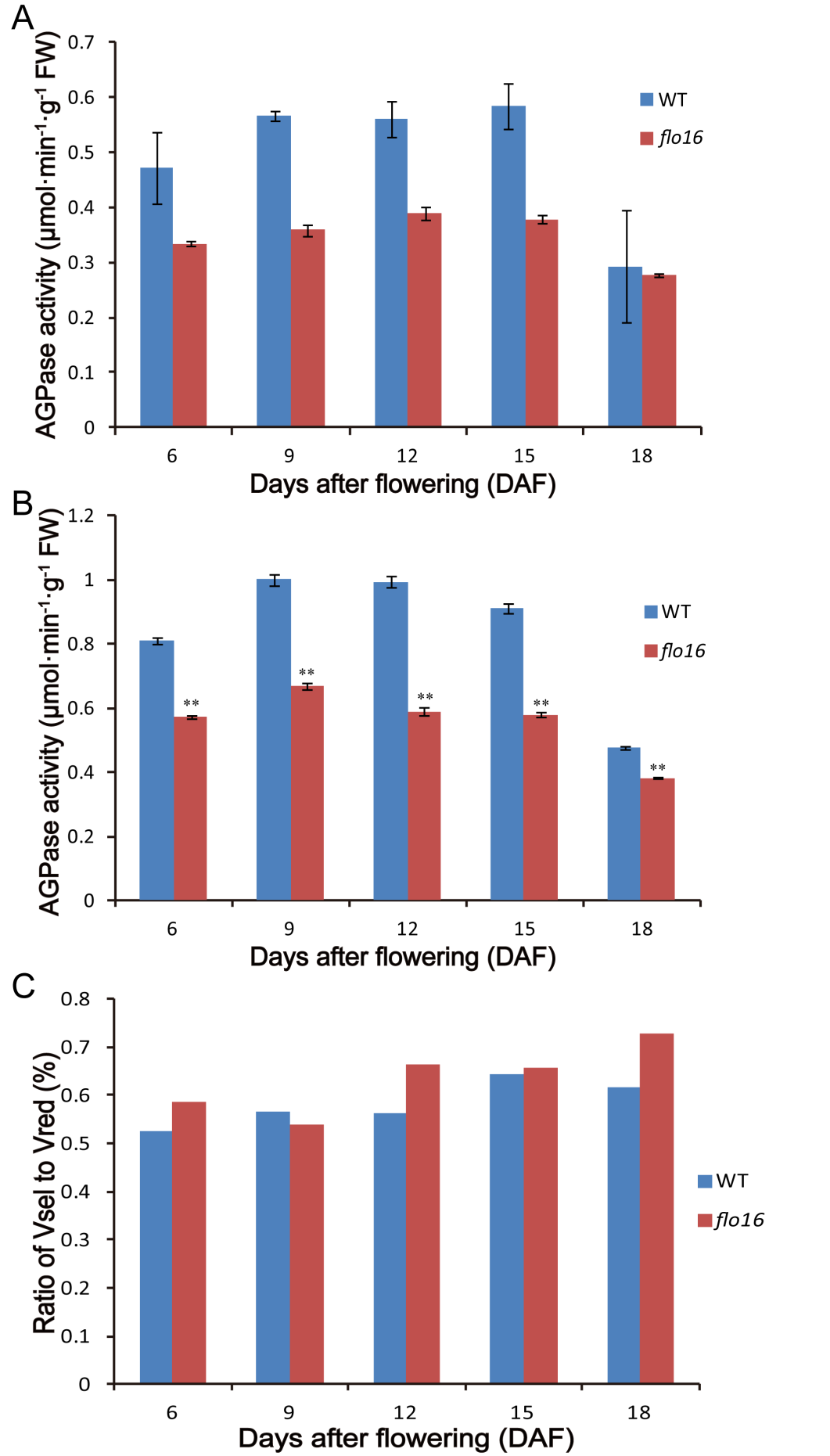
*

**Figure S4.** Redox activation state of AGPase. (B) Overall AGPase activity when 5mM DTT was absent in the assay (Vsel). Values are means ± SD, n = 3. (B) Overall AGPase activity when 5mM DTT was included in the assay (Vred). Values are means ± SD, n = 3. (C) Redox activation state of AGPase (Vsel/Vred). Vsel indicates native AGPase activity without DTT in the assay.


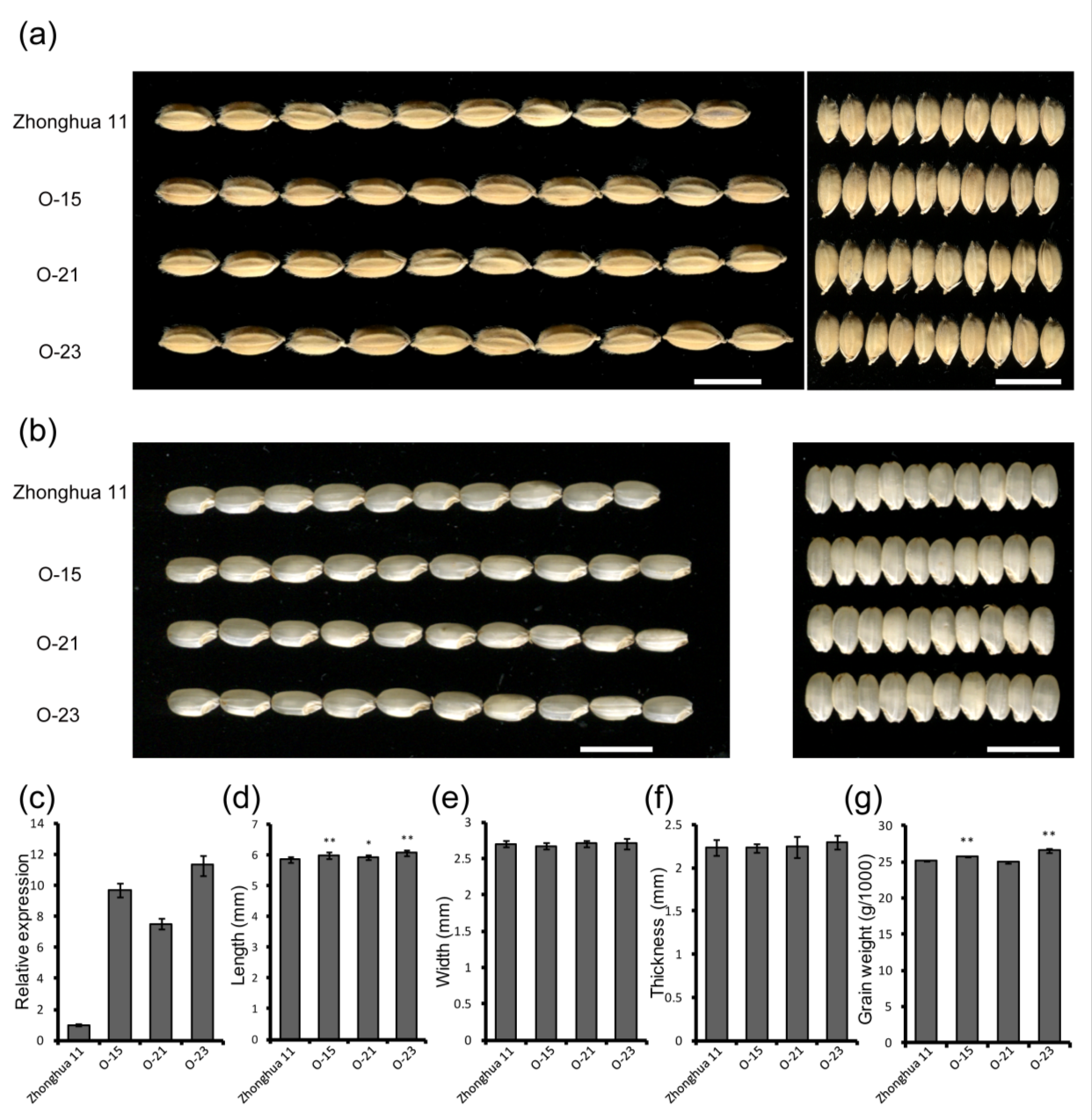

**Figure S5.** Effects of *FLO16* over-expression on grains. (a, b) Images of hulled (a) and dehulled (b) seeds of the recipient and transgenic lines. O-15, O-21 and O-23 are independent transgenic lines. Scale bars, 1 cm. (c) Enhanced expression levels of *FLO16* in over-expression lines. Values are means ± SDs, n = 3. (d-f) Size comparisons between the wild type and transgenic seeds. Values are means ± SDs, n = 10. (g) 1000 grain weights of overexpression lines. Values are means ± SDs, n = 3. Asterisks indicate the statistical significance between the wild type and the mutant, determined by Student’s *t*-tests (*P <0.05; **P <0.01).

**Table S1.** Comparison of agronomic traits between the wild type and *flo16* mutant.

|  | WT | *flo16* |
| --- | --- | --- |
| Young leaf length (cm) | 12.79 ± 1.07 | 8.35 ± 1.70** |
| Young root length (cm) | 8.20 ± 0.71 | 7.39±1.35* |
| Plant height (cm) | 154.70 ± 8.38 | 149.75 ± 6.32* |
| Heading period (d) | 91 ± 3 | 94 ± 4* |
| Panicle length (cm) | 24.0 ± 1.9 | 21.2 ± 2.5** |
| Tilling number | 11 ± 4 | 7 ± 2** |
| Grains per panicle | 144 ± 23 | 127 ± 22* |
| Seed setting (%) | 92.72 ± 4.16 | 94.93 ± 2.76 |

Values are means ± SD, n = 20. Asterisks indicate the statistical significance between the wild type and the mutant, as determined by a Student’s *t-*test (*P <0.05; **P <0.01).

**Table S2.** Agronomic traits of overexpression lines.

|  | Zhonghua11 | EO-27 | EO-29 | EO-31 |
| --- | --- | --- | --- | --- |
| Plant height (cm) | 107.22 ± 2.71 | 105.72 ± 2.32 | 105.53 ± 1.95* | 107.18 ± 4.26 |
| Main panicle length (cm) | 24.32 ± 0.93 | 24.52 ± 1.25 | 23.09 ± 0.87** | 22.33 ± 0.96** |
| Tilling number | 8.52 ± 2.92 | 11 ± 4.54 | 10.32 ± 3.92 | 10 ± 2.48 |
| Grains per main panicle | 199.18 ± 23.56 | 186.43 ± 12.87 | 191.38 ± 22.09 | 194.5 ± 12.92 |
| Seed setting (%) | 76.96 ± 4.8 | 75.32 ± 6.5 | 71.7 ± 3.81 | 73.23 ± 8.69 |

Values are means ± SD, n = 10. Asterisks indicate the statistical significance between the wild type and the mutant, as determined by a Student’s *t-*test (*P <0.05; **P <0.01).

**Table S3.** Oligonucleotide primers used in map-based cloning.

| Marker | Forward primer | Reverse primer |
| --- | --- | --- |
| I10-6 | AATGACAAGGCCGACGATAG | TATTACCCAGGCCAACCTGT |
| 10-24 | CTACACGCGCAAACTCTGTC | ATGAAGGTCTAGGCTGCACC |
| 10-26 | CCAGCTGTTAATTAGCATGTGTGAGC | CAGCAAAGGCGTACGTATCTCG |
| H188-2 | AGTCATCATAAGCGAAGGA | CATGGTTACGATGGCTCT |
| 188-5 | TCTGACGAAGTACACCTCCTG | GTCCCAAACCTCTAAATGAATA |
| H188-15 | TTGATTACAACCAACTCTGACC | GGCCCTTACCCATTGTA |
| 188-21 | TTTTGATGATGCCTACTCCA | GTAATTTGAAAAGCGTGCC |
| 188-2 | GTGTTCGTCCAAAGAAAT | CCTAGCTGCAAGTAAATC |

**Table S4.** Gene-specific primers used in this study.

| Purpose | Primer name | Primer sequence (5’ to 3’) |
| --- | --- | --- |
| qRT-PCR | FLO16-RT-F | GACTCCCAGTGGAGAGAAGC |
|  | FLO16-RT-R | CTGCTGGACGGTAGAGATGA |
|  | Actin-F | CCCTCCTGAAAGGAAGTACAGTGT |
|  | Actin-R | GTCCGAAGAATTAGAAGCATTTCC |
| Subcellular localization | FLO16-GFP-F | GCCCAGATCAACTAGTATGGCGAAGGAACCGATGCGC |
|  | FLO16-GFP-R | TGCTCACCATGGATCCGTTGAGGCATGAGTAAGCG |
| Functional complementation | FLO16-W-F | CCGGCGCGCCAAGCTTAAGTAATTTGGGAAAGAGG |
|  | FLO16-W-R | GAATTCCCGGGGATCCTTAGTTGAGGCATGAGTAAG |
| Endosperm-specific expression | FLO16-E-F | GGGGTACCATGGCGAAGGAACCGATGCGC |
|  | FLO16-E-R | GGACTAGTTTAGTTGAGGCATGAGTAA |
| Over-expression | FLO16-O-F | TTACTTCTGCACTAGGTACCATGGCGAAGGAACCGATGCGC |
|  | FLO16-O-R | GAATTCCCGGGGATCCTTAGTTGAGGCATGAGTAA |
| CRISPR | FLO16-C-F | GGCATGCGATCACATTCGTGAC |
|  | FLO16-C-R | AAACGTCACGAATGTGATCGCA |
